# Supplementary material for: Association of serum 25-Hydroxy vitamin D with total and regional adiposity and cardiometabolic traits
Source: PLoS One. 2020 Dec 28;15(12):e0243850. doi: 10.1371/journal.pone.0243850 (PMC7769464; doi:10.1371/journal.pone.0243850)
Supplement: S1 Table — (DOCX) [file pone.0243850.s001.docx]

|  | **Group** | **Men** | **P value** | **Women** | **P value** |
| --- | --- | --- | --- | --- | --- |
| Urban^b^ | Studied  [n=373] | 105 (51.2) | 0.61 | 86 (51.2) | 0.825 |
|  | Not studied  [n=707] | 186 (49.5) |  | 164 (49.5) |  |
| Age(years) | Studied  [n=373] | 41.4 (1.0) | 0.82 | 41.4 (1.0) | 0.245 |
|  | Not studied  [n=707] | 41.4 (0.9) |  | 41.5 (1.1) |  |
| BMI(kg/m^2^) | Studied  [n=373] | 24.9 (4.4) | 0.17 | 26.1 (5.2) | 0.248 |
|  | Not studied  [n=707] | 24.4 (4.1) |  | 26.7 (5.2) |  |
| Waist Circumference(cm) | Studied  [n=373] | 90.9 (12.3) | 0.18 | 83.7 (12.0) | 0.282 |
|  | Not studied  [n=707] | 89.5 (11.5) |  | 84.9 (12.1) |  |
| Hip Circumference(cm) | Studied  [n=373] | 91.7 (8.7) | 0.08 | 95.5 (10.9) | 0.484 |
|  | Not studied  [n=707] | 90.5 (7.7) |  | 96.2 (11.3) |  |
| Physical activity score ^a^ | Studied  [n=373] | 1200  (845 -1568.5) | 0.15 | 1723.9  (1367.7-2119.1) | 0.344 |
|  | Not studied  [n=707] | 1162.5  (795.0-1430.7) |  | 1677.8  (1357.9-2031.4) |  |
| SES (Household possession score)^b^  1  2  3  4 | Studied  [n=373] | 54 (26.4)  46 (22.4)  56 (27.3)  49(23.9) | 0.58 | 51 (30.4)  36 (21.4)  40 (23.8)  41 (24.4) | 0.774 |
| 1  2  3  4 | Not studied  [n=707] | 101 (26.9)  94 (25.0)  84 (22.3)  97 (25.8) |  | 98 (29.6)  65 (19.6)  93 (28.1)  75 (22.7) |  |
| Total Fat(kg) ^a^ | Studied  [n=373] | 14.9 (10.9-19.3) | 0.10 | 20.8 (16.3-25.9) | 0.260 |
|  | Not studied  [n=707] | 14.1 (9.9-18.3) |  | 21.6 (16.9-26.7) |  |
| Android Fat(kg) ^a^ | Studied  [n=373] | 1.4 (0.9-1.8) | 0.11 | 1.4 (0.9-2.0) | 0.367 |
|  | Not studied  [n=707] | 1.3 (0.8-1.7) |  | 1.5 (1.0-1.9) |  |
| Gynoid Fat(kg) ^a^ | Studied  [n=373] | 2.4 (1.8-3.0) | 0.11 | 3.5 (2.9-4.3) | 0.44 |
|  | Not studied  [n=707] | 2.3 (1.8-2.8) |  | 3.6 (2.9-4.4) |  |
| Visceral fat (kg) ^a^ | Studied  [n=373] | 0.4 (0.3-0.6) | 0.39 | 0.3 (0.2-0.5) | 0.68 |
|  | Not studied  [n=707] | 0.4 (0.3-0.5) |  | 0.3 (0.2-0.5) |  |
| Leg fat(kg) ^a^ | Studied  [n=373] | 2.7 (1.9-3.5) | 0.17 | 4.3 (3.3-5.2) | 0.30 |
|  | Not studied  [n=707] | 2.5 (1.8-3.3) |  | 4.40 (3.6-5.4) |  |
| Total lean muscle mass (kg) ^a^ | Studied  [n=373] | 54.3 (47.6-58.9) | 0.79 | 38.9 (35.0-44.9) | 0.09 |
|  | Not studied  [n=707] | 52.8 (47.9-59.0) |  | 40.9 (36.4-46.0) |  |
| Systolic BP (mmHg) | Studied  [n=373] | 127.0 (16.4) | 0.64 | 118.6 (13.4) | 0.82 |
|  | Not studied  [n=707] | 127.7 (15.9) |  | 118.3 (13.8) |  |
| Diastolic BP (mmHg) | Studied  [n=373] | 80.2 (12.9) | 0.90 | 75.8 (9.7) | 0.64 |
|  | Not studied  [n=707] | 80.4 (12.5) |  | 76.2 (10.0) |  |
| HDL - cholesterol (mmol/l) | Studied  [n=373] | 1.05 (0.3) | 0.44 | 1.15 (0.2) | 0.23 |
|  | Not studied  [n=707] | 1.1 (0.3) |  | 1.2 (0.3) |  |
| LDL – cholesterol (mmol/l) | Studied  [n=373] | 3.00 (0.8) | 0.57 | 2.9 (0.8) | 0.90 |
|  | Not studied  [n=707] | 2.9 (0.8) |  | 2.9 (0.7) |  |
| Total Cholesterol (mmol/l) | Studied  [n=373] | 4.7 (1.1) | 0.46 | 4.5 (0.9) | 0.46 |
|  | Not studied  [n=707] | 4.5 (0.9) |  | 4.4 (0.8) |  |
| Triglycerides (mmol/L)^a^ | Studied  [n=373] | 1.6 (1.0-2.3) | 0.56 | 1.1 (0.7-1.5) | 0.91 |
|  | Not studied  [n=707] | 1.4 (1.0-2.2) |  | 1.0 (0.8-1.5) |  |
| Fasting plasma glucose (mmol/L) ^a^ | Studied  [n=373] | 5.4 (5.0-5.9) | 0.014 | 5.3 (4.9-6.0) | 0.48 |
|  | Not studied  [n=707] | 5.2 (4.8-5.8) |  | 5.3 (4.9-5.8) |  |
| Fasting insulin (mlU/ml)^a^ | Studied  [n=373] | 8.1 (4.5-12.9) | 0.06 | 7.6 (4.5-11.8) | 0.20 |
|  | Not studied  [n=707] | 6.7 (3.1-13.6) |  | 7.0 (3.8-10.9) |  |
| HOMA-IR^a^ | Studied  [n=373] | 2.0 (1.1-3.3) | 0.046 | 1.9 (1.0-3.2) | 0.14 |
|  | Not studied  [n=707] | 1.7 (0.7-3.3) |  | 1.7 (0.9-2.9) |  |
| HOMA-B^a^ | Studied  [n=373] | 86.1 (42.8-124.4) | 0.56 | 76.0(46.8-98.9) | 0.65 |
|  | Not studied  [n=707] | 71.6 (48.8-113.0) |  | 72.8(51.8-99.4) |  |
| Impaired fasting glucose^b^ | Studied  [n=373] | 47 (22.9) | 0.59 | 43 (25.6) | 0.81 |
|  | Not studied  [n=707] | 79 (21.0) |  | 88 (26.6) |  |
| Type 2 diabetes^b^ | Studied  [n=373] | 43 (21.0) | 0.44 | 31 (18.5) | 0.05 |
|  | Not studied  [n=707] | 69 (18.4) |  | 40 (12.1) |  |
| Hypertension^b^ | Studied  [n=373] | 45 (22.0) | 0.69 | 12 (7.1) | 0.40 |
|  | Not studied  [n=707] | 88 (23.4) |  | 31 (9.4) |  |
| Hypertriglyceridemia^b^ | Studied  [n=373] | 92 (44.9) | 0.57 | 28 (16.7) | 0.20 |
|  | Not studied  [n=707] | 178 (47.3) |  | 71 (21.5) |  |

**Supplementary Table 1. Sensitivity analysis comparing individuals studied vs. not studied (without 25(OH)D measurements)**

Values are mean (SD) for normally distributed variables and p value is obtained from t test. ^a^median (interquartile range) for skewed variables and p value is obtained from non-parametric Mann Whitney test; ^b^n (%) for categorical variables and p value is obtained from Pearson Chi-square tests**.**
